# Inferior vena cava isolation for non–pulmonary vein–origin atrial fibrillation identified via self-reference mapping using a high-density grid catheter

**DOI:** 10.1016/j.hrcr.2025.08.015

**Published:** 2025-08-19

**Authors:** Yasuteru Yamauchi, Yuichiro Sagawa, Kazuya Murata, Fumitaka Kikuchi, Tetsuo Sasano, Kazutaka Aonuma

**Affiliations:** 1 Department of Cardiology, Japan Red Cross Yokohama City Bay Hospital, Kanagawa, Japan; 2 Hachinoe Heart Center Clinic, Aomori, Japan; 3 Department of Cardiology, Institute of Science Tokyo, Tokyo, Japan; 4 Department of Cardiology, Mito Saiseikai General Hospital, Ibaraki, Japan

**Keywords:** Inferior vena cava, Isolation, Atrial fibrillation, Non–pulmonary vein, Self-reference mapping, High-density grid catheter, Exit block

**Key Teaching Points**

• Self-reference mapping with a high-density grid catheter enables the accurate localization of atrial fibrillation (AF) triggers within the inferior vena cava (IVC), which are difficult to detect using conventional mapping tools.
• Frequent ectopic discharges with an exit block revealed a functional conduction boundary between the IVC and right atrium, defining an arrhythmogenic substrate within the IVC.
• Linear ablation along the IVC-right atrial boundary achieved complete electrical SVC isolation and resulted in long-term AF elimination, thus offering a robust strategy for managing IVC-origin AF.

## Case report

**Figure 1:**
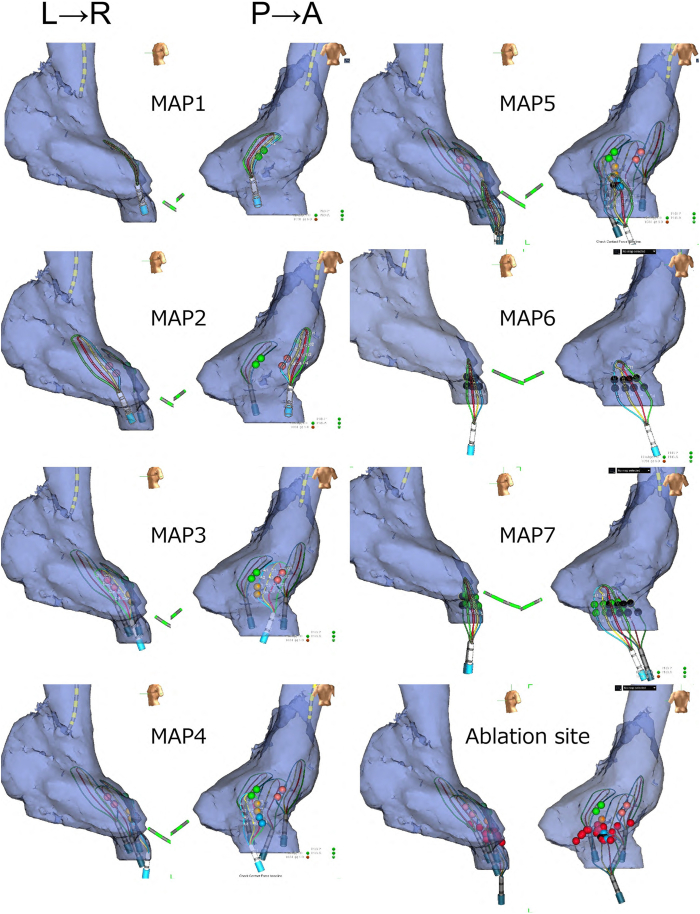
Identification of non–pulmonary vein (PV) foci via self-reference mapping using a high-density (HD) grid catheter. When non-PV focus was initiated, the earliest excited site on the HD grid catheter was tagged. The *green tag* in MAP1 indicates the earliest activation, suggesting that tachycardia originates from the posterior or lateral wall of the right atrium. We then moved the HD grid catheter toward the lateral wall; similarly, the *red tag* in MAP2 indicates the earliest activation, reinforcing the posterior wall. When the HD grid catheter was positioned between MAP1 and MAP2, the *yellow tag* marked the earliest site, suggesting that the origin of tachycardia was the lower part of the posterior wall. Further mapping with the lower HD grid catheter revealed the earliest activation in the inferior vena cava–right atrial junction, indicated using a *blue tag*. Finally, after slightly retracting the HD grid catheter and tagging the earliest activation site with a *black tag*, the location was found to almost perfectly overlap with the previously marked *blue tag*, confirming the origin of the tachycardia at this precise site. When the HD grid catheter was slightly adjusted to the side, abnormal excitations accompanied by an exit block were observed at the *black and green tag points* in MAP6 and MAP7. The final ablation sites are indicated with *red tags*. L→R = left-to-right view; P→A = posterior-to-anterior view.

**Figure 2:**
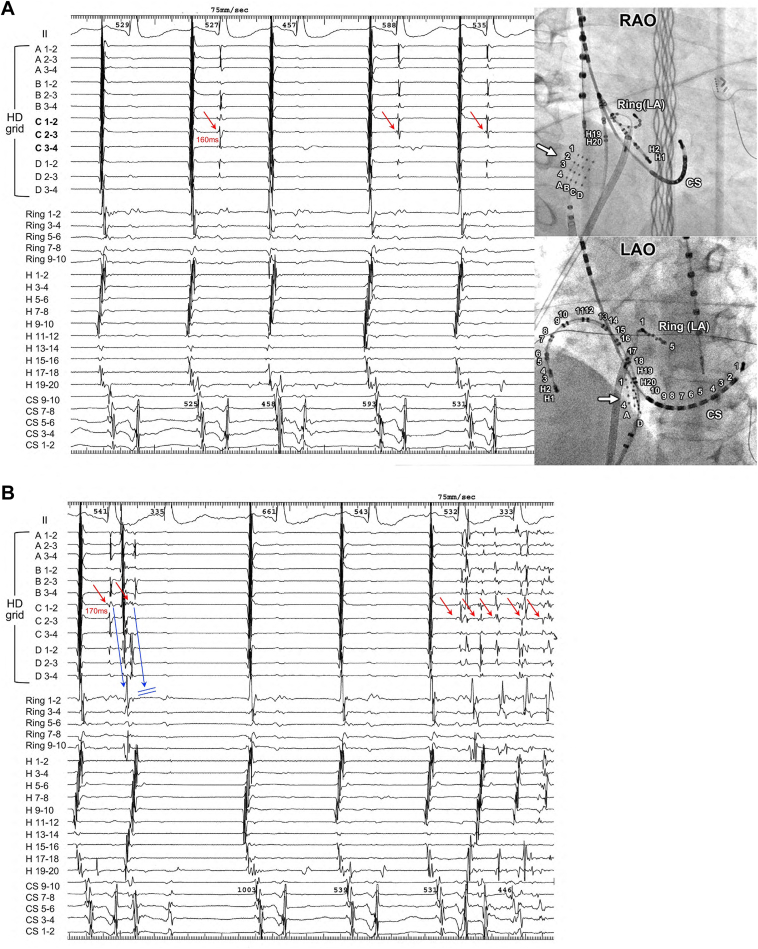
Abnormal excitations with an exit block. Intracardiac electrograms and angiograms during abnormal excitations with an exit block were recorded using a high-density grid catheter positioned within the inferior vena cava (IVC) (**A**). Intracardiac electrograms were recorded during the onset of atrial fibrillation (**B**). The first abnormal excitation had a 170 ms coupling interval, which was 10 ms longer than that of the exit block. The first abnormal excitation was conducted to the right atrium (RA), whereas the second abnormal excitation, which exhibited a conduction block at the RA-IVC junction, failed to propagate to the RA. Eventually, the clustering of abnormal excitations led to the induction of AF. CS = coronary sinus; H = halo catheter; LAO = left anterior oblique; RAO = right anterior oblique; Ring = a ring catheter positioned at the left atrial septum.

**Figure 3:**
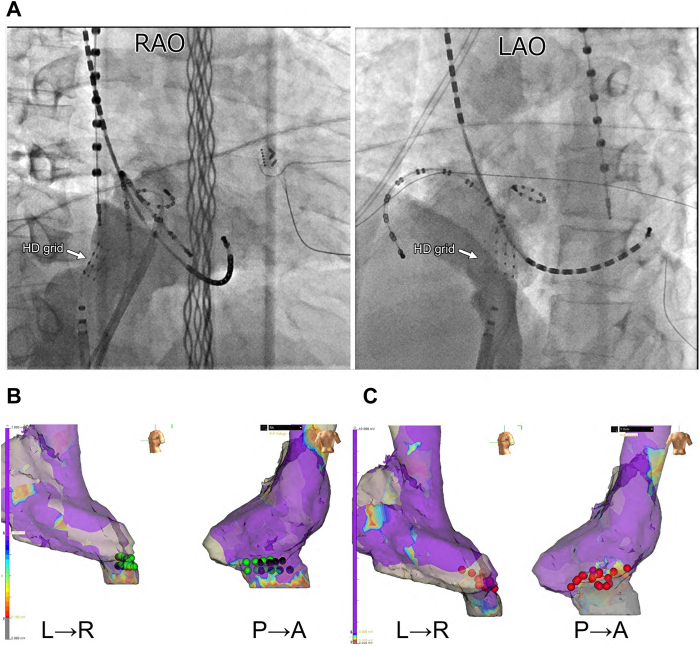
Venograms and voltage maps before and after ablation. Venograms illustrating the anatomy of the inferior vena cava (IVC) and the right atrium highlight the positioning of the high-density (HD) grid catheter within the IVC (**A**). Voltage maps recorded before and after ablation are shown. All *green and black tags* indicate sites within the IVC where abnormal potentials associated with localized conduction block between the IVC and right atrium were recorded using the HD grid catheter (**B**), whereas *red tags* mark the ablation sites (**C**). After linear ablation at the posteroseptal border between the right atrium and IVC, the IVC potentials disappeared, indicating complete electrical isolation of the IVC. LAO = left anterior oblique; L→R = left-to-right view; P→A = posterior-to-anterior view; RAO = right anterior oblique.

A 70-year-old man was referred for catheter ablation of paroxysmal atrial fibrillation (AF). Echocardiography revealed a normal ejection fraction and left atrial diameter of 38 mm. First, pulmonary vein (PV) isolation and cavotricuspid isthmus ablation were performed using an irrigated-tip radiofrequency ablation catheter. After ablation, non-PV-origin AF was induced via isoproterenol infusion. After direct-current cardioversion, non-PV-origin AF was reinitiated. We then positioned a wide halo catheter with duodecapolar electrodes throughout the right atrium and a ring catheter in the left atrial septum to simultaneously map both sides of the atrial septum. Despite repeated electrical cardioversion, AF recurred with identical atrial activation sequences, with the earliest atrial activation site located in the lower right atrial septum. Consequently, we performed non-PV trigger mapping with self-reference mapping using a high-density (HD) grid catheter to obtain a more detailed map (Figure 1, MAP1–5). Finally, we identified the precise origin of non-PV triggers within the inferior vena cava (IVC). Notably, abnormal single ectopic beats with a short coupling interval of 160 ms, which did not conduct to the right atrium, were observed during sinus rhythm (Figure 1, MAP6 and MAP7; Figure 2A). Furthermore, we observed that when the coupling interval of the abnormal excitation was slightly prolonged, it propagated to the right atrium. Subsequently, burst excitations originating from the IVC emerged and degenerated into the AF (Figure 2B). Contrast imaging of the right atrium via the IVC revealed that the HD grid catheter was positioned on the posterior wall at the anatomic boundary between the right atrium and IVC (Figure 1, MAP6 and 7; Figure 3A). Using an HD grid catheter, we tagged the sites where abnormal excitations showed an exit block, a conduction block between the right atrium and the IVC. These sites were recorded on the voltage map of the right atrium and IVC (Figure 3B). The locations tagged with green and black dots, as shown in Figure 3B, are believed to electrophysiologically correspond to areas where sleeve-like myocardial extensions of the IVC exist, given that they exhibit a functional conduction block between the IVC and right atrium. Radiofrequency energy was delivered using an open-irrigated contact force–sensing catheter (TactiCath SE, Abbott) at 25–30 W for 30 seconds per application during linear ablation along the electroanatomic junction between the IVC and right atrium, resulting in successful IVC isolation and elimination of non-PV-origin AF (Figure 1, MAP8; Figure 3C). To assess dormant conduction, 20 mg of adenosine triphosphate was administered as a bolus injection at the end of the procedure; however, no reconduction was observed. Since the procedure, the patient has remained under follow-up for 4 years with no recurrence of AF.

## Discussion

Non-PV-origin AF triggers are often found in the superior vena cava (SVC), atrial septum, or left atrial posterior wall.^1^ However, rare frequent sites, such as the vein of Marshall, atrial appendages, and IVC, have also been documented.^2^ It is sometimes challenging to accurately identify the origin of non-PV triggers. Advanced mapping techniques, such as self-reference mapping using an HD grid catheter, have considerably improved the ability to localize non-PV triggers.^3^^,^^4^ By detecting and tagging the earliest activation sites with an HD grid catheter and progressively tracing upstream activations, this method identifies overlapping activation points, enabling precise localization of non-PV triggers. In the present case, self-reference mapping revealed that the arrhythmogenic focus was located in the IVC. Abnormal excitation with a conduction block between the IVC and right atrium was observed, allowing clear delineation of the electrophysiological boundary between the IVC and right atrium. This conduction barrier is a distinct feature, marked by abnormal impulses confined to the IVC and electrically dissociated from right atrial activity. Scavee et al^5^ reported the detection of ectopic discharges within the IVC that were not detected in the right atrium using a ring catheter. However, their study did not use 3-dimensional voltage mapping; therefore, details such as the extent of the IVC tissue exhibiting these abnormal excitations could not be obtained. Consequently, focal ablation was performed at the earliest site of excitation within the IVC. These findings suggest the presence of an autonomous arrhythmogenic substrate within the IVC. The use of an HD grid–mapping catheter facilitated the precise characterization of this substrate, providing critical information for effective intervention.

Igawa et al^6^ reported variability in the extent of IVC projections into the posteroinferior region of the right atrium. The IVC typically enters the right atrium at an oblique angle, and a low-voltage area is often observed in the posteroinferior region of the right atrium. Importantly, the anatomic boundary between the IVC and the right atrium may differ from the boundary perceived on simple fluoroscopic images. Moreover, Hashizume et al^7^ noted that, histologically, the IVC shares similar arrhythmogenic potential with the thoracic veins, including the PVs and SVC, owing to the extension of the cardiac musculature from the right atrium in humans. However, the myocardial sleeve of the IVC is considerably shorter than that of the SVC. In addition, the IVC is considered electrically less active than the SVC, largely because of the frequent scarcity of longitudinal myocardial fibers.

Recently, Nie et al^8^ reported that 6 of 661 patients (0.91%) had paroxysmal AF triggered by IVC. According to their findings, the average distance from the earliest activation site to the IVC ostium was 6.8 ± 2.5 mm (range 5.2–11.2 mm), with a total of 2.3 ± 0.5 radiofrequency energy applications used to eliminate the IVC triggers. In addition, all arrhythmogenic foci within the IVC were located in the apical hemisphere, with 3 on the septal and 3 on the anterior side. However, they noted a limitation in that the 3 cases of triggers originating from the anterior wall of the IVC could not be strictly distinguished anatomically from those originating from the Eustachian ridge. Only instances considered to be purely IVC-origin AF were of septal origin. In this case, the AF trigger originated from the posterior septum and was associated with an exceptionally broad IVC sleeve myocardium. In all previously reported cases of IVC-origin AF, focal ablation was successful, likely because the IVC sleeve was limited in size. However, in cases in which the IVC sleeve myocardium is exceptionally broad, focal ablation may pose a risk of AF recurrence. Based on these findings, linear ablation was performed along the boundary between the IVC and right atrium to achieve complete electrical IVC isolation. Unlike focal ablation that targets specific ectopic sites, this comprehensive strategy addresses the substrate and ensures a durable outcome. Although IVC isolation poses potential risks such as IVC stenosis or phrenic nerve injury owing to the anatomic proximity of critical structures, several factors suggest that the actual risk in this case was minimal. First, unlike full circumferential ablation, our approach involved limited linear ablation, reducing the likelihood of IVC stenosis. In addition, SVC isolation, which is a widely performed procedure, has been reported to rarely result in SVC stenosis. Based on these considerations, we proceeded with IVC isolation as a safe and anatomically guided strategy for eliminating non-PV triggers. This innovative approach represents a notable progress in the treatment of IVC-origin AF by addressing the arrhythmogenic substrate at its source and reducing the likelihood of AF recurrence.

Suzuki et al^9^ reported a case of localized AF within the inferior sinus venosa successfully treated by isolation, which bears similarity to our present case. Although both cases involve ablation near the junction between the IVC and the right atrium, key differences exist. In our case, frequent abnormal excitations confined to the IVC with clear exit block allowed precise delineation of the boundary between the IVC and the right atrium using HD self-reference mapping, a definition not previously reported. In contrast, Suzuki et al^9^ described the arrhythmogenic region as the “inferior sinus venosa,” reflecting uncertainty about whether it originated from IVC tissue or adjacent right atrium, and did not demonstrate exit block confined to IVC potentials or complete dissociation from right atrial activity. This electrophysiological distinction highlights the novelty and mechanistic insight of our case.

This case demonstrates the feasibility of IVC isolation for eliminating non-PV triggers. However, given that this is a single case report, the generalizability and reproducibility of this approach remain limited. Further studies involving larger cohorts and multicenter experiences are needed to validate its safety and efficacy. In addition, future research should focus on defining appropriate selection criteria, procedural endpoints, and long-term outcomes to better clarify the clinical role of IVC isolation.

## Conclusion

The presented case highlights the effectiveness of advanced self-reference mapping using an HD grid catheter in precisely localizing non-PV-origin AF triggers. Frequent abnormal excitation of the IVC with an exit block facilitated clear delineation of the electrical boundary between the IVC and right atrium. Linear ablation along this boundary achieved effective electrical isolation of the IVC, resulting in sustained long-term elimination of AF. Thus, this approach represents a notable advancement in the management of IVC-origin AF, providing durable outcomes and expanding treatment paradigms.

## Disclosures

The authors have no conflicts of interest to disclose.

## References

[1] Takigawa M., Takahashi A., Kuwahara T. (2015). Impact of non-pulmonary vein foci on the outcome of the second session of catheter ablation for paroxysmal atrial fibrillation. J Cardiovasc Electrophysiol.

[2] Santangeli P., Zado E.S., Hutchinson M.D. (2016). Prevalence and distribution of focal triggers in patients with persistent and long-standing persistent atrial fibrillation. Heart Rhythm.

[3] Matsunaga-Lee Y., Takano Y. (2018). A novel mapping technique to detect non–pulmonary vein triggers: a case report of self-reference mapping technique. Heart Rhythm Case Rep.

[4] Yamauchi Y., Nakamura R., Shigeta T., Sagawa Y., Okishige K., Sasano T. (2021). Persistent atrial fibrillation originating from prominent eustachian ridge: precise identification of non-pulmonary vein foci using a high-density grid mapping catheter. Heart Rhythm Case Rep.

[5] Scavee C., Jais P., Weerasooriya R., Haïssaguerre M. (2003). The inferior vena cava: an exceptional source of atrial fibrillation. J Cardiovasc Electrophysiol.

[6] Igawa O., Adachi M., Yano A. (2006). Extension of the inferior vena cava into the posteroinferior right atrium. Heart Rhythm.

[7] Hashizume H., Ushiki T., Abe K. (1995). A histological study of cardiac muscles of the human superior and inferior vena cava. Arch Histol Cytol.

[8] Nie Z., Chen S., Lin J. (2022). Inferior vena cava as a trigger for paroxysmal atrial fibrillation: incidence, characteristics, and implications. JACC Clin Electrophysiol.

[9] Suzuki Y., Akita T., Takami K., Ishihata T., Yoshida A. (2025). Localized atrial fibrillation within the inferior sinus venosa treated by isolation: could it be a new target of ablation for persistent atrial fibrillation?. Heart Rhythm Case Rep.

